# Hydrogel Scaffold Printing Assisted by Substrate Cooling and an Electric Field

**DOI:** 10.3390/polym18202579

**Published:** 2026-10-20

**Authors:** Wenbo Jin, Ruoyu Wang, Wenyu Ning, Fei Duan, Liangkun Lu, Cheng Zhang, Heqi Xu, Kedong Song, Danyang Zhao

**Affiliations:** 1 State Key Laboratory of High-Performance Precision Manufacturing, Dalian University of Technology, Dalian 116024, China; jinwenbo19940820@163.com (W.J.); ruoyu@mail.dlut.edu.cn (R.W.); nwy20010304@mail.dlut.edu.cn (W.N.); df5981483@163.com (F.D.); chengz9106@163.com (C.Z.); kedongsong@dlut.edu.cn (K.S.); zhaody@dlut.edu.cn (D.Z.); 2 Ningbo Institute of Dalian University of Technology, Ningbo 315032, China; 3 School of Mechanical and Electrical Engineering, Zhengzhou University of Light Industry, Zhengzhou 450002, China

**Keywords:** hydrogel printing, substrate cooling, electric-field-assisted extrusion, pore geometry, multilayer scaffolds

## Abstract

Lateral spreading and deformation during layer stacking limit the geometric fidelity of extrusion-printed hydrogel scaffolds. To address these constraints, this study presents an extrusion-printing strategy that combines electrical assistance and substrate cooling for poly(vinyl alcohol)/gelatin methacryloyl/alginate hydrogels. Electrical assistance improved filament uniformity in the simulations and independent controls, while separate cooling controls showed better preservation of lattice openings. The process produced filaments approximately 152 μm wide, and connected patterns retained distinct corners, curved boundaries and junctions. Three independently printed grids yielded a square-pore printability index of 0.9256 ± 0.0034. Three ten-layer specimens also retained open pores and reached a mean height of approximately 1.40 mm. After printing, ultraviolet irradiation and three freeze–thaw cycles stabilized the constructs. Compression tests on cast hydrogel specimens showed increasing resistance to deformation with additional freeze–thaw cycles. Curved patterns, structures of different heights and a meniscus-shaped construct further demonstrated the range of printable geometries. Together, these results support the strategy for fine filament deposition and open multilayer construction in this hydrogel blend.

## 1. Introduction

The geometry of an extrusion-printed hydrogel scaffold depends on the deposition and stabilization of successive filaments. A continuous strand can spread laterally after contacting the substrate, narrowing the intended gaps between neighboring paths. At intersections, material accumulation changes the shape of the pore corners; during stacking, deformation of the lower layers alters the final height. These changes make filament formation, pore geometry and multilayer retention complementary measures of printability [1]. Rheological assessments relate extrusion and recovery to stable deposition, while filament-fusion and collapse tests examine ink responses to path spacing and support conditions [2,3]. Controlling the transition from a flowing precursor to a shape-retaining strand is therefore central to reproducing a designed scaffold architecture.

In addition to ink properties, the deposition environment provides a means of controlling this transition. Freeform reversible embedding of suspended hydrogels (FRESH) uses a temporary support medium to hold soft material as the structure is printed [4]. Shear-thinning inks can likewise be deposited into self-healing hydrogel supports [5]. In granular gels, local fluidization permits nozzle motion, while recovery of the surrounding medium supports the deposited path [6]. By providing external support, these approaches can preserve geometries that would otherwise deform during construction. For printing onto an exposed platform, however, stabilization must develop at the substrate and within the deposited layers. This makes the interval between material arrival and shape fixation particularly important for retaining the printed geometry.

Electrical assistance addresses an earlier stage by guiding filament delivery before substrate contact. Electrohydrodynamic printing uses electrically induced flow to guide material through fine nozzles and form patterned deposits [7]. Through functional-ink formulation and jetting control, this approach has also been developed for flexible electronics [8]. Examples include highly ordered poly(3-hexylthiophene) nanowires for flexible organic field-effect transistors and metal–quartz composite nozzle designs for stable printing on flexible substrates [9,10]. Electrical deposition on a cooled collector has enabled polycaprolactone scaffold fabrication, while cryo-electrohydrodynamic jetting has produced porous constructs from aqueous silk fibroin solutions [11,12]. In this combination, the electric field influences the emerging material, while collector temperature affects its behavior after deposition. For hydrated polymer blends, the resulting geometry also depends on crosslinking of the deposited precursor and subsequent treatment.

Cryogenic printing has been used to build frozen structures in a liquid-nitrogen bath and soft poly(vinyl alcohol) (PVA)-containing hydrogels on a cooled substrate [13,14]. Embedded cryogel printing has combined gelatin methacryloyl (GelMA)/alginate deposition in a calcium-containing nanoclay bath with ultraviolet (UV) crosslinking after freezing [15]. Multimaterial cryogenic printing has further coordinated freezing, solvent melting and crosslinking to construct complex hydrogel architectures [16]. These approaches share two connected stages: thermal fixation supports geometry during fabrication, while polymer-network formation contributes to the stability of the processed material. This relationship is especially relevant when thin deposited paths must remain separated through repeated layer addition.

In this study, we developed a pneumatic extrusion strategy that combines electrical guidance before deposition with cooling-assisted fixation and post-print stabilization to preserve fine, open geometries in PVA/GelMA/sodium alginate (ALG) hydrogels. Numerical simulations describe electrically assisted filament formation, while independent controls distinguish the electrical and cooling contributions to filament uniformity and pore preservation. Rheological characterization relates precursor composition to extrusion and filament retention, and compression tests on cast hydrogels assess freeze–thaw stabilization. Temperature-dependent pore analysis and multilayer height measurements quantify geometric fidelity. Curved paths, variable-height structures and a meniscus-shaped construct extend the strategy beyond regular lattices, providing a geometric proof of concept for fabricating complex hydrogel architectures.

## 2. Material Characterization and Printing Methodology

### 2.1. Precursor Preparation and Material Characterization

The aqueous precursor contained 5 wt% PVA, 4 wt% GelMA, 1.5 wt% ALG, 0.05 wt% calcium chloride and 0.25 wt% Irgacure 2959. Concentrations were expressed relative to the total precursor mass, with deionized water as the solvent. PVA (molecular weight 89,000–98,000 g/mol; ≥99% hydrolyzed) was obtained from Sigma-Aldrich, ALG from Aladdin and Irgacure 2959 from Macklin. Irgacure 2959 was used as the photoinitiator. All reagents were used without further purification.

**Figure 1:**
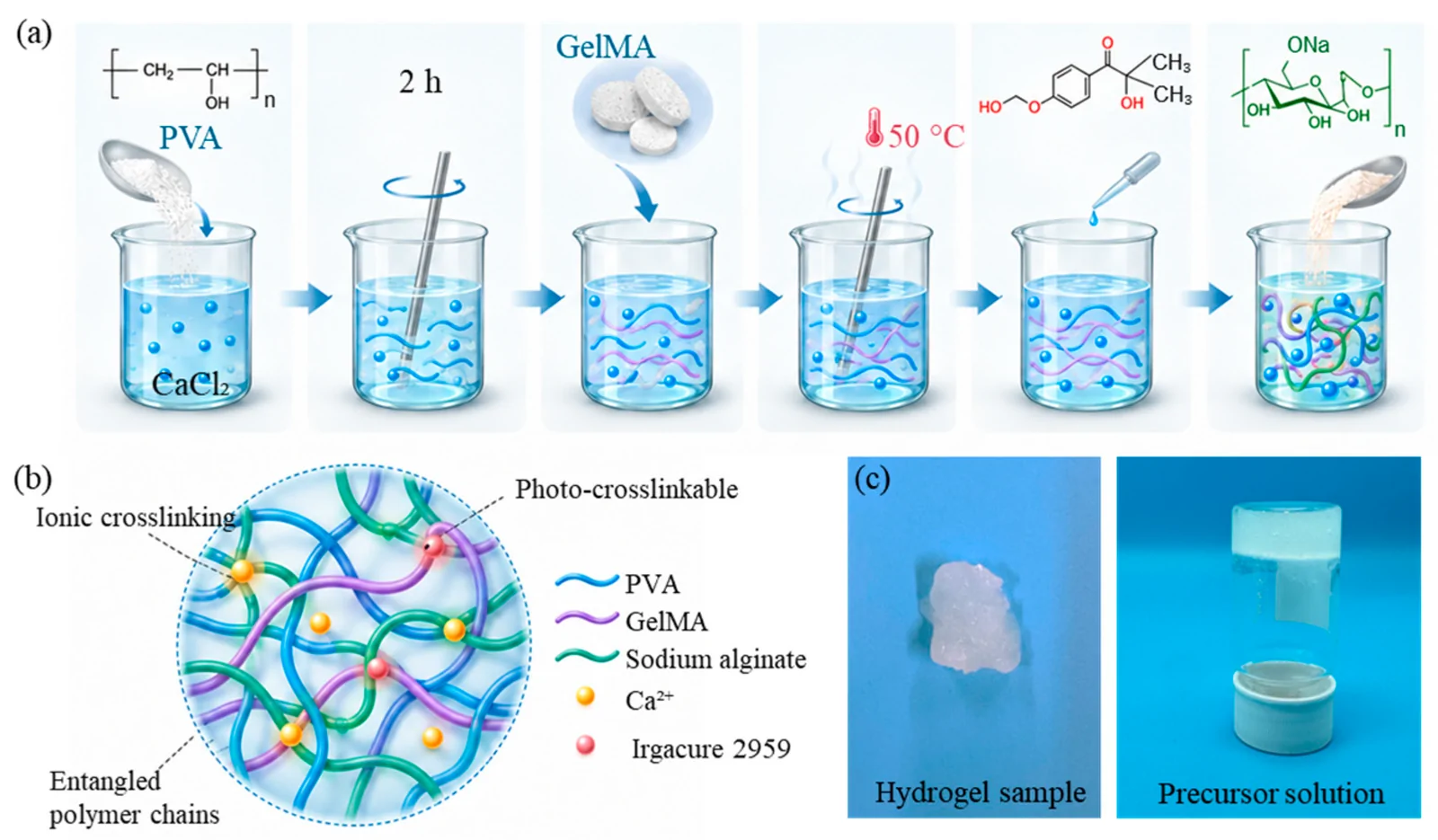
Precursor preparation and hydrogel appearance. (**a**) Preparation sequence. (**b**) Proposed polymer interactions. (**c**) Hydrogel specimen (**left**) and precursor solution (**right**).

PVA was gradually dispersed in the aqueous calcium chloride solution at room temperature. The dispersion was heated to 80 °C and stirred for 2 h until transparent. After cooling to 50 °C, GelMA was added and stirred until dissolved. The mixture was then cooled to 40 °C, followed by the addition of Irgacure 2959 and a further 30 min of stirring. ALG was introduced at room temperature and mixed under mild stirring for 1 h. The resulting precursor was vacuum-degassed for 15 min and loaded into sterile 1 mL glass syringes. Figure 1a summarizes the preparation sequence. Figure 1b identifies the polymer chains, calcium ions and photoinitiator alongside the proposed interactions within the blend. The liquid precursor and treated hydrogel illustrate the transition from a flowable solution to a specimen that retains its external shape (Figure 1c). This transition involves the network-forming mechanisms of the PVA/GelMA/ALG blend during UV exposure and subsequent freeze–thaw treatment. The methacryloyl groups in GelMA provide a route to network formation through photoinitiated crosslinking after printing [17]. Irradiation conditions influence the crosslinking kinetics and resulting material properties [18]. Repeated freezing and thawing can introduce crystalline junctions into PVA hydrogels, while calcium ions can form ionic connections between alginate chains [19,20].

**Figure 2:**
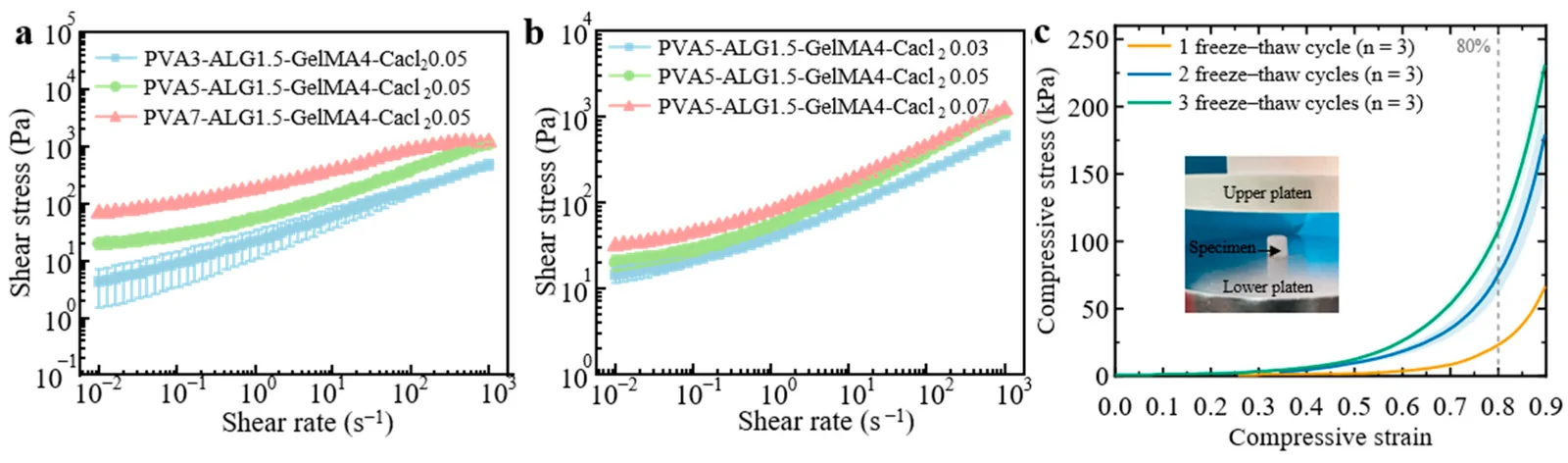
Rheological and compressive characterization. (**a**) PVA contents of 3, 5 and 7 wt%. (**b**) CaCl_2_ contents of 0.03, 0.05 and 0.07 wt%. (**c**) Compressive stress–strain curves after one, two and three freeze–thaw cycles. Curves show mean values; error bars and shaded bands represent SD (n = 3).

Rheological measurements were performed at 25 °C using a rheometer (HR20) with 20 mm parallel plates and a 0.5 mm gap. Shear-rate sweeps covered 0.01–1000 s^−1^ with logarithmically spaced points, and yield stress was determined by fitting the shear stress–shear rate curves to the Herschel–Bulkley model [21]. PVA contents of 3, 5 and 7 wt% were tested at fixed 1.5 wt% ALG, 4 wt% GelMA and 0.05 wt% CaCl_2_, while CaCl_2_ contents of 0.03, 0.05 and 0.07 wt% were tested at fixed 5 wt% PVA, 1.5 wt% ALG and 4 wt% GelMA. Three independently prepared samples were tested per formulation. All formulations exhibited shear thinning and composition-dependent yield stresses (Figure 2a,b). Increasing PVA from 3 to 7 wt% raised the yield stress from 1.171 to 34.34 Pa, consistent with increased polymer-chain entanglement and flow resistance. The 5 wt% PVA formulation exhibited an intermediate yield stress of 17.07 Pa. Increasing CaCl_2_ from 0.03 to 0.07 wt% raised the yield stress from 10.61 to 23.68 Pa, consistent with stronger Ca^2+^-mediated alginate associations. Shear thinning facilitates extrusion, while yield stress helps resist deformation after deposition. Together with the printing observations, these results support 5 wt% PVA and 0.05 wt% CaCl_2_ for balancing extrusion resistance and filament retention. Cast cylinders, 10 mm in diameter and height, received the UV treatment described in Section 2.3 and one, two or three freeze–thaw cycles. Compression tests were performed using a testing machine (DR-607A, Dongguan Dongri Instrument Co., Ltd., China) at 2 mm/min (n = 3). Compressive stress increased with freeze–thaw cycles, with the three-cycle group showing the highest stress at a given strain (Figure 2c). This trend is consistent with reinforcement of the PVA physical network. Specimen height recovered from 8.5 mm immediately after unloading to 9.7 mm after 10 min, approaching the initial height of 10.0 mm and indicating substantial dimensional recovery.

### 2.2. Printing Principle, Numerical Simulation and Model Validation

**Figure 3:**
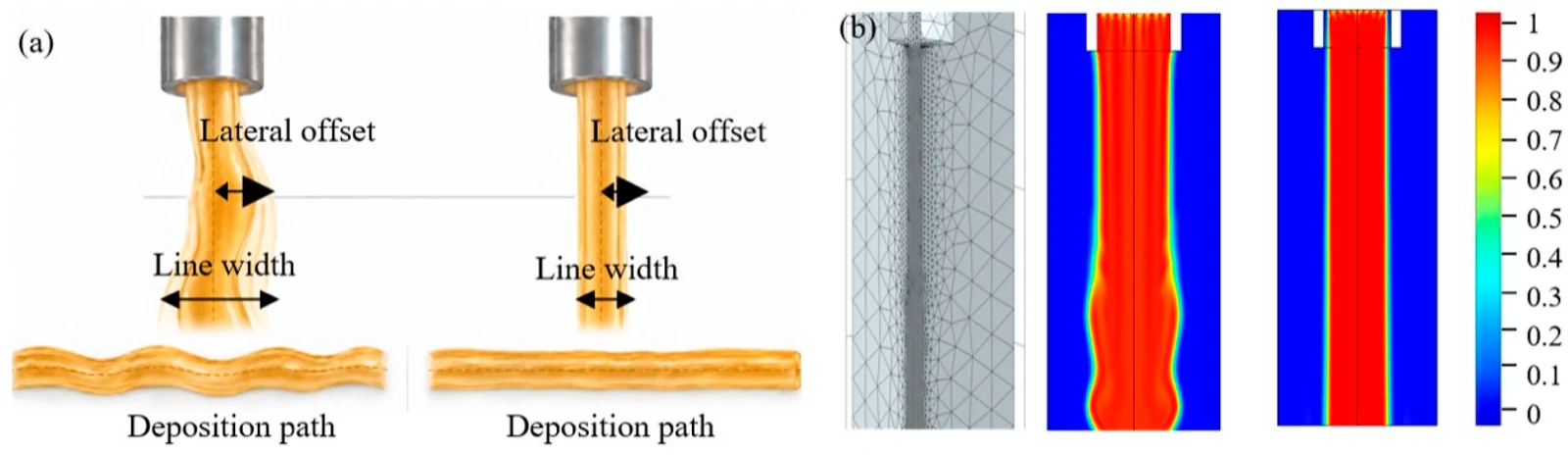
Printing concept and two-phase simulation. (**a**) Deposition without (**left**) and with (**right**) electrical assistance. (**b**) Computational mesh (**left**) and phase volume-fraction distributions without (**middle**) and with (**right**) electrical assistance.

The schematic compares filament delivery and deposition paths with and without electrical assistance (Figure 3a). Pneumatic pressure drives the precursor through the needle, while the applied potential difference introduces electrical stress between the printhead and collector. Electrical stress can direct the emerging material toward the substrate, with viscous and capillary stresses also affecting its free-surface shape. Numerical simulations were performed in COMSOL Multiphysics 6.1 (COMSOLAB, Stockholm, Sweden) using a two-dimensional Cartesian model with a 150 μm nozzle opening. The incompressible Navier–Stokes equations were coupled to the Cahn–Hilliard phase-field equation and electrostatics. Electrical body forces were introduced through Fx and Fy, with phase-fraction-dependent permittivity. The isothermal model included gravity and surface tension.

The two-dimensional, isothermal model used a constant effective viscosity of 1.75 Pa·s to compare filament formation with and without electrical assistance under fixed material properties. The precursor density was 1120 kg/m^3^, and the surface tension was 60 mN/m. Viscosity, density and surface tension were measured by rheometry, pycnometry and tensiometry, respectively. A pressure of 150 kPa was applied at the inlet, and a no-slip condition was imposed on the nozzle walls. The nozzle potential was set to 0 or 1.2 kV, the bottom boundary was grounded, and the remaining electrostatic boundaries used zero charge. The electric field was calculated from the imposed potential as E = −∇V. The simulation used a single mesh of 25,080 triangular elements, locally refined near the nozzle and filament. Transient solutions used adaptive backward differentiation formula (BDF) integration with a relative tolerance of 0.005.

Figure 3b shows the mesh and phase volume-fraction distributions on a dimensionless 0–1 scale. The simulated filament without electrical assistance showed alternating bulges and constrictions along its axial contour. With electrical assistance, the contour was straighter and the liquid-phase region varied less in width along the nozzle axis. At 1.2 kV and 150 kPa, the simulated filament width was 150 μm. Printing at 150 mm/min with the same voltage and pressure yielded a measured filament width of 152 μm (relative difference, 1.32%). The width comparison applies to the tested condition; predicting cooled deposition requires consideration of temperature- and shear-dependent rheology, heat transfer, phase change and three-dimensional effects.

### 2.3. Printing Platform and Stabilization Procedure

**Figure 4:**
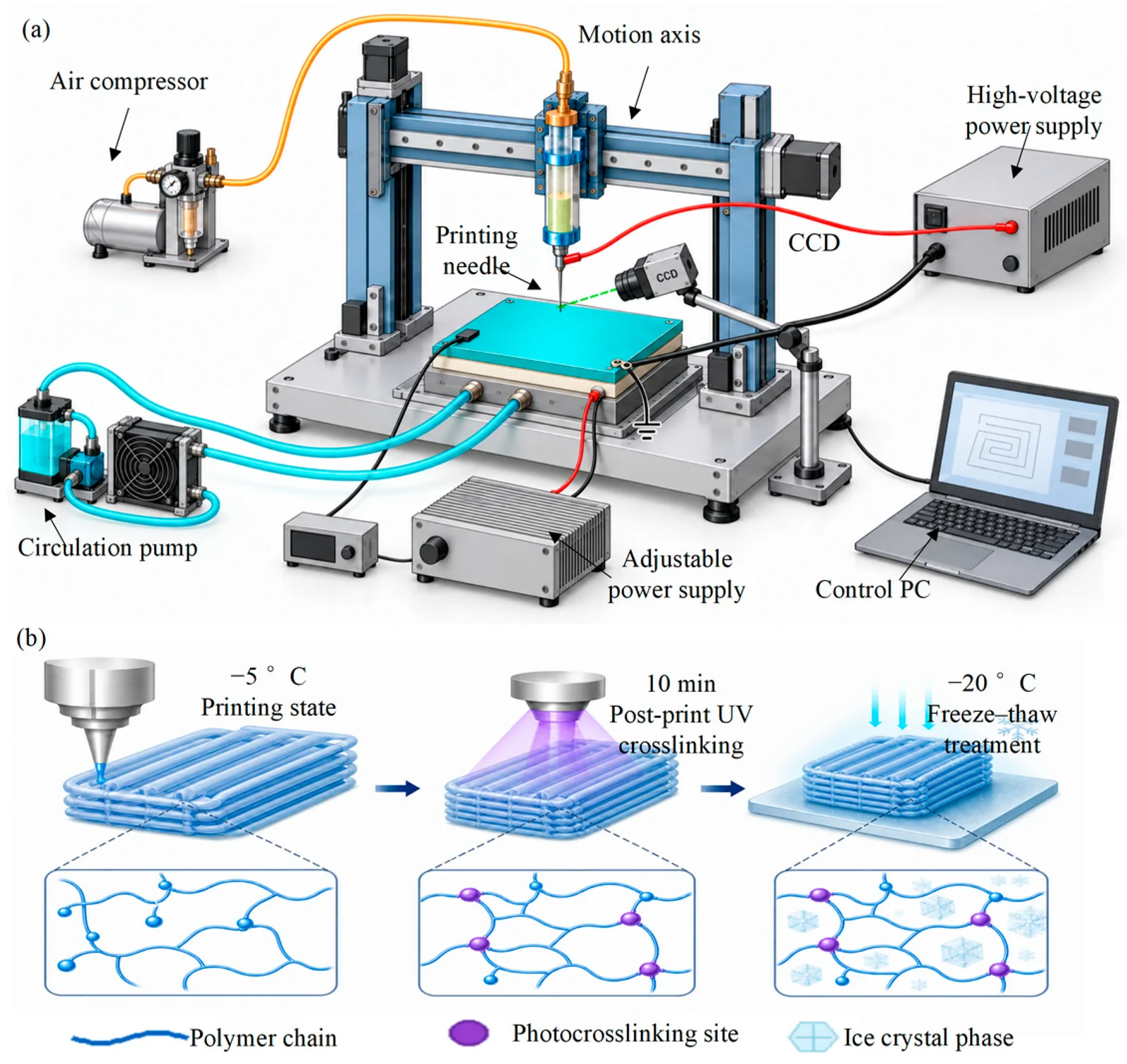
Printing system and stabilization sequence. (**a**) Experimental setup. (**b**) Cooling-assisted deposition and post-print treatment.

The printing platform integrated pneumatic extrusion, a computer-controlled three-axis motion system, a high-voltage power supply and a cooled collector (Figure 4a). A 1 mL glass syringe supplied the precursor through a metallic needle with an internal diameter of 150 μm, with compressed air providing the extrusion pressure. The motion system controlled the nozzle trajectory and interlayer displacement to fabricate prescribed planar paths and multilayer structures. The high-voltage lead was connected to the metallic printhead assembly, and the return lead was connected to the grounded platform. This configuration established a potential difference across the nozzle-to-platform gap during extrusion.

A thermoelectric module beneath the collector controlled the substrate temperature. An adjustable power supply drove the module, while a circulation pump supplied coolant to the heat-removal circuit. Heat was extracted from the precursor on contact with the collector and from subsequent deposits through the underlying layers. The combined printing condition used 1.2 kV, 150 kPa, 150 mm/min and a platform temperature of −5 °C. Voltage, pneumatic pressure and printing speed were set for each experiment, while the nozzle-to-platform distance was maintained at 2 mm. An optical camera monitored the nozzle–substrate region to track lateral displacement, interruptions in material delivery and accumulation along the deposited path. The UV lamp remained off during printing.

The stabilization sequence links cooling during deposition to polymer-network formation after printing (Figure 4b). Cooling extracts heat from the aqueous precursor, while ice formation can restrict liquid redistribution and temporarily support the deposited shape. Partial freezing can also concentrate polymer in the remaining unfrozen regions. For an open lattice, timely shape fixation limits spreading into neighboring openings and preserves the pore boundaries inherited by subsequent layers. Support-assisted collagen printing illustrates how control of material setting can preserve complex shapes during fabrication [22]. This stabilization complements electrical guidance of the emerging filament before substrate contact. After each construct was completed, the GelMA-containing precursor was photocrosslinked using a 365 nm UV LED source for 10 min. The irradiance at the sample was 10 mW/cm^2^, and the source-to-sample distance was 10 cm. The constructs then underwent three freeze–thaw cycles, each comprising freezing at −20 °C for 8 h and thawing at room temperature for 2 h, to further develop the hydrogel network.

### 2.4. Printing Conditions and Experimental Design

Single-filament and letter-pattern specimens were printed under two conditions: 0 kV at 25 °C and 1.2 kV at −5 °C. Both used a needle internal diameter of 150 μm, a pneumatic pressure of 150 kPa and a printing speed of 150 mm/min. The nozzle-to-platform distance was 2 mm. To separate electrical and cooling effects, filaments were printed at 0 and 1.2 kV with the platform held at 25 °C. Lattices were printed at 25 and −5 °C with the voltage held at 0 kV. Both control series used 100 kPa, 3 mm/s, a 150 μm needle and a 2 mm nozzle-to-platform distance. Three specimens were printed independently for each condition. The controls evaluated individual effects under their stated conditions; the interaction between electrical assistance and cooling was not quantified.

For the temperature series, the platform was set to 25, 10, 0 or −5 °C, with voltage, pneumatic pressure and printing speed held at 1.2 kV, 150 kPa and 150 mm/min, respectively. Multilayer specimens were printed under the combined printing condition through the 150 μm needle in ten layers with a nominal total height of 1.5 mm. The constructs received the UV and freeze–thaw treatment described in Section 2.3. Optical images were acquired during low-temperature printing and after crosslinking to examine the deposited paths and processed constructs. The tested conditions were not systematically optimized; a broader process window requires assessment of precursor composition, nozzle geometry, deposition speed and cooling.

### 2.5. Geometric Measurements and Statistical Analysis

Pore boundaries were extracted using ImageJ 1.54p with the Fiji distribution [23,24]. The dimensionless square-pore printability index was calculated as $\mathrm{Pr}\;=\;\frac{L^{2}}{16A}$, where *L* is the pore perimeter and *A* is the enclosed area [25]. An ideal square has Pr = 1. Pr characterizes pore shape; filament-width variation, dimensional accuracy and structural uniformity are complementary measures of printing fidelity. Three specimens were printed independently per condition for the straight-filament controls, lattice controls, temperature series and ten-layer height measurements. The three prints per condition were technical replicates from the same ink batch on the same day, assessing consistency within that batch. Three filaments were examined per straight-filament specimen. At −5 °C, 25 pores were analyzed in each of three independently printed grids. Each ten-layer specimen was measured at five positions on its ImageJ side-view profile. A mean was first calculated for each specimen. Geometric group results were reported as the mean ± sample standard deviation (SD) of the three specimen means (n = 3).

### 2.6. Optical and Scanning Electron Microscopy

Optical microscopy was used to examine filament outlines, lattice openings and multilayer profiles. Scanning electron microscopy (SEM; JSM-7610F, JEOL Ltd., Akishima, Tokyo, Japan) was used to observe filament junctions and finer surface morphology. After the post-print treatment described in Section 2.3, the SEM specimen was freeze-dried for 24 h and gold-coated before imaging. SEM imaging was performed at an accelerating voltage of 3.0 kV and a working distance of 15.5 mm. Overview and enlarged images were acquired from the same specimen at magnifications of 25× and 100×, respectively.

## 3. Printing Results and Discussion

### 3.1. Filament Uniformity and Lattice Definition

**Figure 5:**
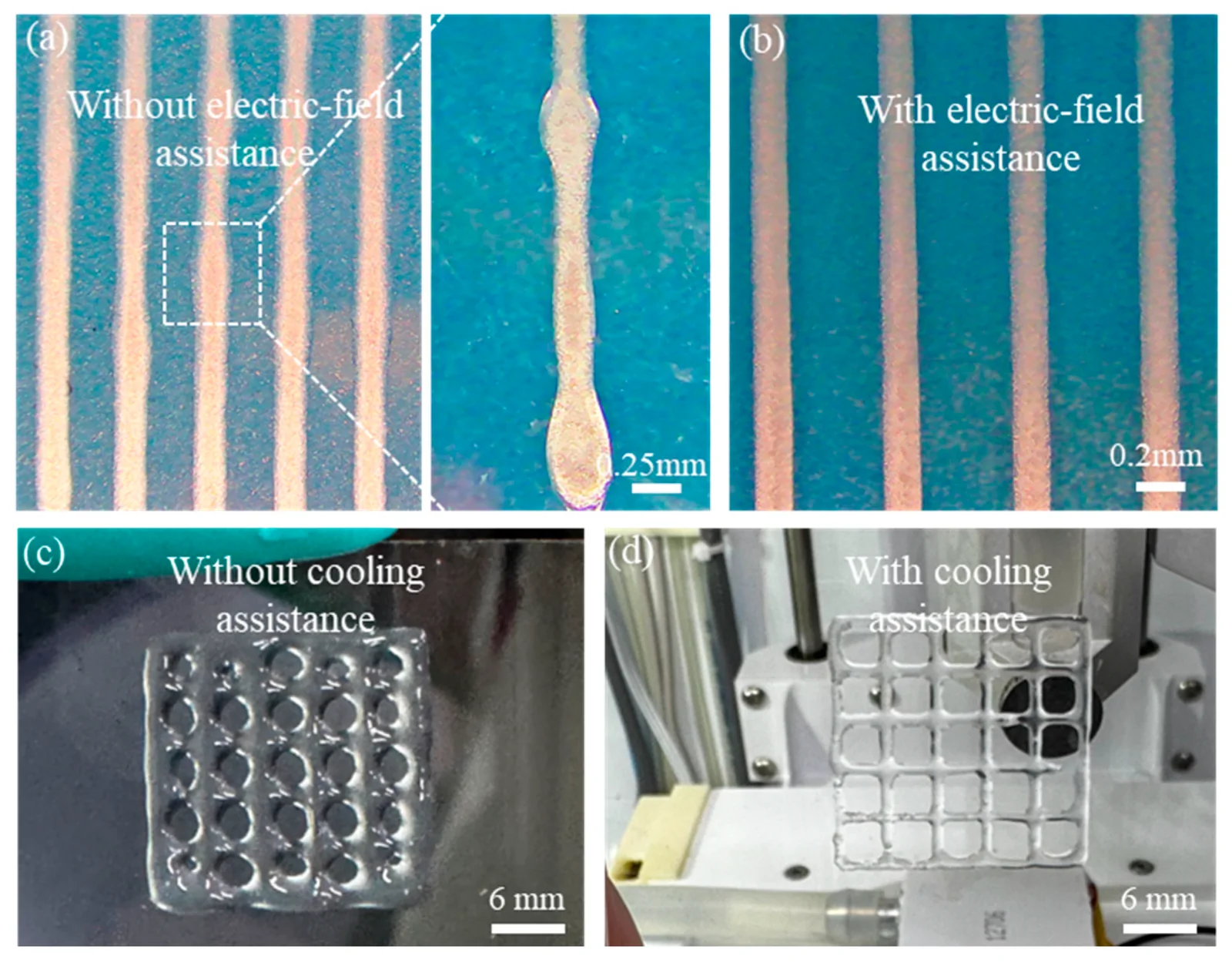
Independent electrical and cooling controls. (**a**,**b**) Filaments at 0 and 1.2 kV, respectively, at 25 °C; (**a**) includes an enlarged view. (**c**,**d**) Lattices at 25 and −5 °C, respectively, at 0 kV.

The deposited strands at 0 kV and 25 °C showed localized enlargements separated by narrower segments (Figure 5a). The enlarged view resolved this bulge–neck–bulge sequence along a continuous filament. At 1.2 kV and 25 °C, electrically assisted strands had more nearly parallel edges and fewer prominent local enlargements (Figure 5b). Adjacent paths remained separated over the imaged length, and the gaps between them were less disrupted by localized accumulation. Substrate cooling improved lattice definition in the 0 kV control (Figure 5c,d). At 25 °C, spreading around strand intersections produced rounded openings and broad junctions. At −5 °C, the grid retained straighter sides, more distinct pore corners and narrower connections between neighboring openings. The combined printing process produced filaments approximately 152 μm wide. These controls identify filament uniformity and pore preservation as distinct outcomes of electrical assistance and substrate cooling.

### 3.2. Temperature-Dependent Pore Formation and Shape Uniformity

**Figure 6:**
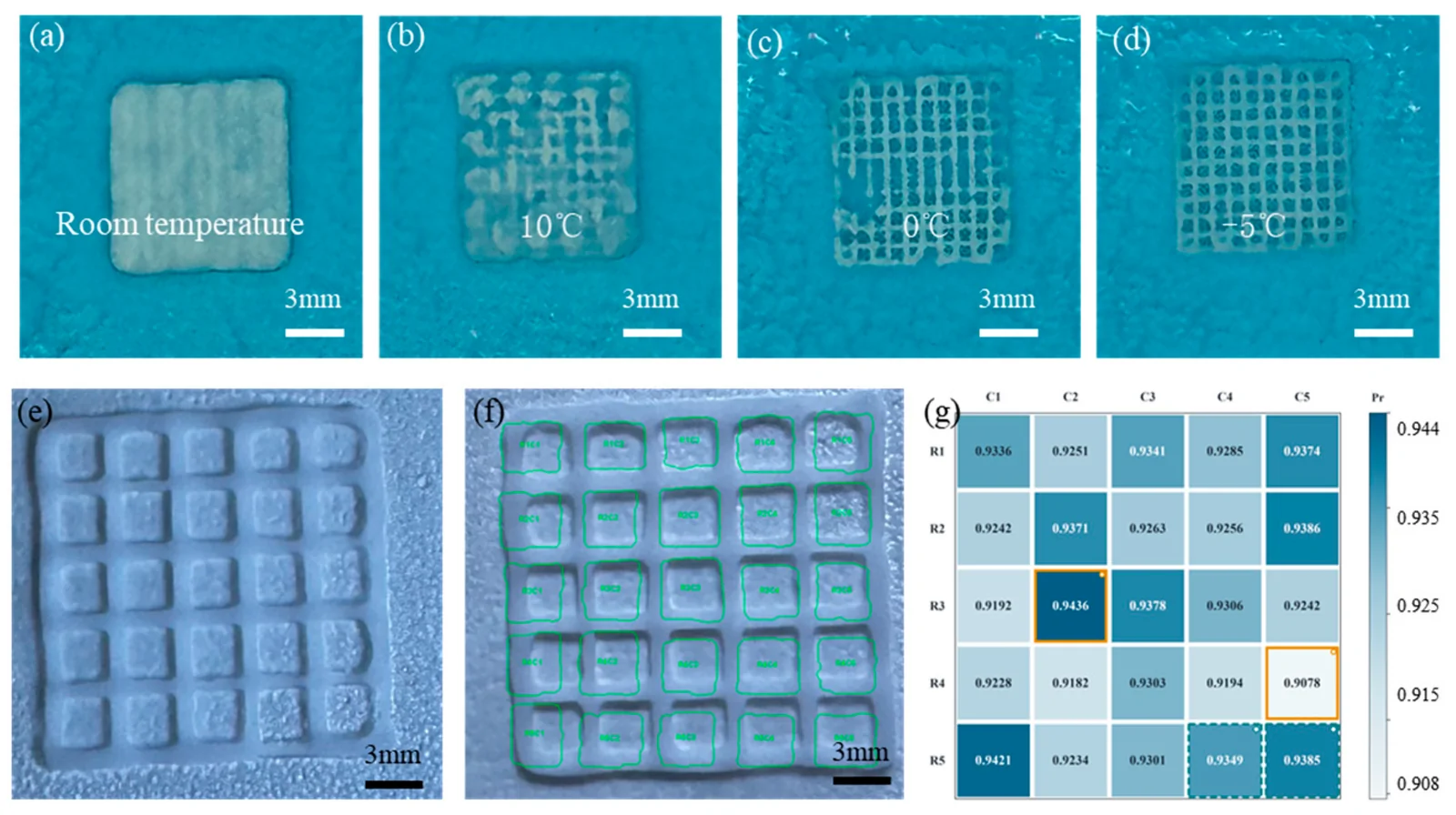
Temperature-dependent lattice formation and pore-shape analysis. (**a**–**d**) Printing at 25, 10, 0 and −5 °C, respectively. (**e**–**g**) Original lattice image, extracted pore contours and spatial distribution of *Pr* within one 25-pore construct. The green outlines in (**f**) indicate the extracted pore boundaries. In (**g**), orange boxes highlight the pores with the highest and lowest Pr values.

Lattice boundaries became progressively clearer as the platform was cooled (Figure 6a–d). At room temperature, the deposited region had broad junctions and poorly resolved boundaries between neighboring paths. At 10 °C, the opening boundaries became clearer, but substantial material remained between them. At 0 °C, rows of openings became distinguishable, although their shapes remained irregular and elongated in parts of the grid. At −5 °C, the openings were more consistently separated and arranged across the printed area. All four conditions used 1.2 kV, 150 kPa and 150 mm/min. For the individual lattices analyzed at 25, 10, 0 and −5 °C, Pr increased from 0.8066 to 0.9046, 0.9155 and 0.9293, respectively. The increase indicated progressively better preservation of square-pore geometry as cooling restricted lateral spreading. Cooling-induced viscosity changes and, at subzero temperature, possible ice formation and polymer concentration in the unfrozen phase may contribute to this stabilization; interfacial thermal history and freezing extent were not measured. Supporting Movie S1 documents deposition and visible shape retention on the cooled substrate.

The imaged lattice contained 25 pores in five rows and five columns. The original image and extracted contours showed approximately straight sides joined by rounded corners (Figure 6e,f). At −5 °C, the mean Pr across three independently printed grids was 0.9256 ± 0.0034 (SD; n = 3). The grid shown in Figure 6e–g had the highest mean Pr (0.9293), and its 25 pores yielded Pr = 0.9293 ± 0.0086 (SD). The rounded corners in the extracted contours were consistent with Pr values below the ideal-square value of 1. Together with the straighter pore sides, they localized the main geometric deviation to strand intersections. The recurring contour may reflect local accumulation at these junctions, consistent with the junction morphology observed in the uncooled lattice. This connection between material delivery and setting was also highlighted in a comparative study of collagen and GelMA that examined extrusion behavior, dimensional stability and photocrosslinking [26]. Temperature-controlled alginate/agar cryoprinting has likewise linked shape retention to gelation, freezing and crosslinking during thawing [27].

### 3.3. Multilayer Shape Retention and Filament Morphology

**Figure 7:**
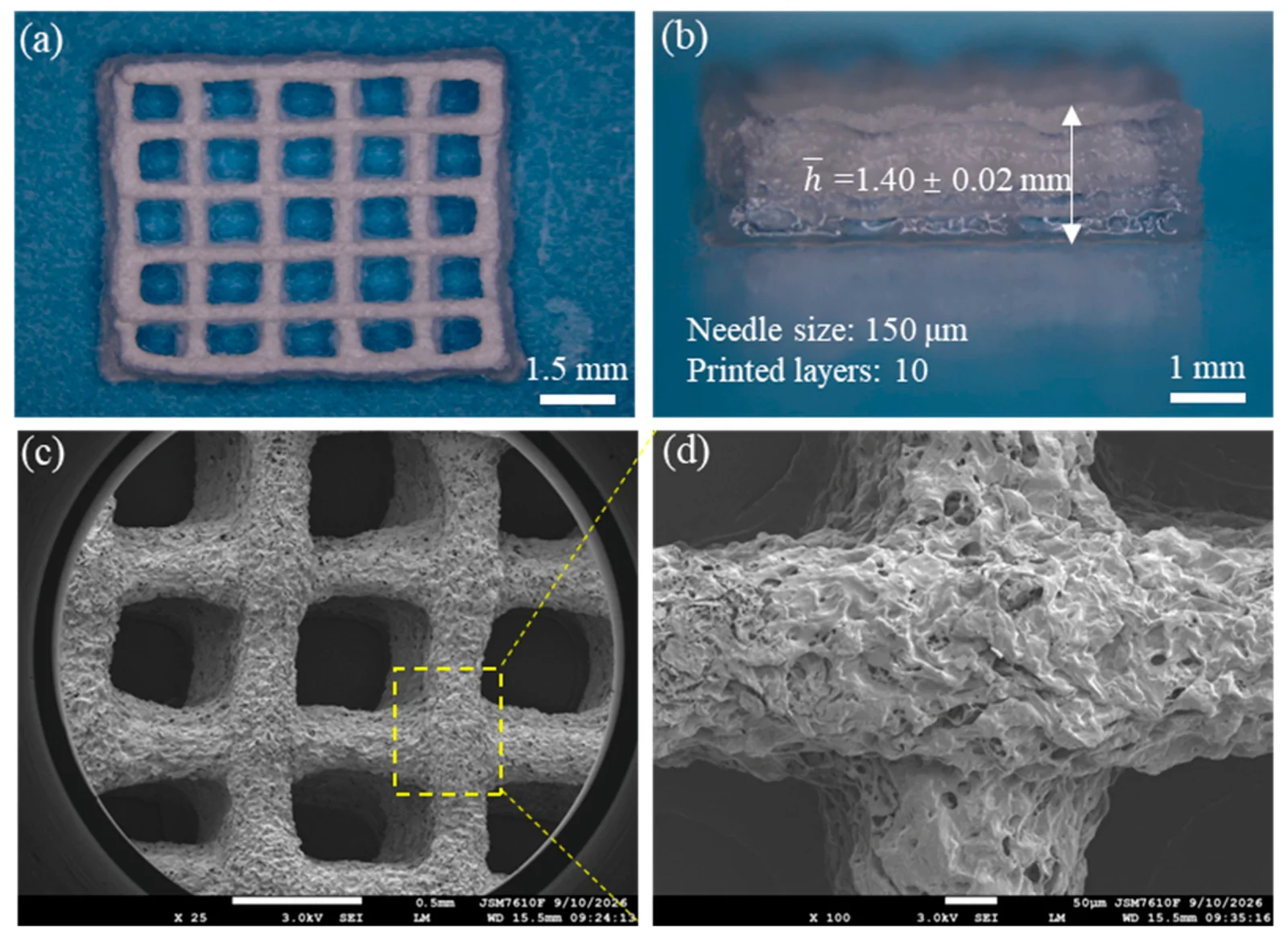
Multilayer geometry and filament morphology. (**a**,**b**) Top and side views of the ten-layer lattice; the height annotation is mean ± SD (n = 3). (**c**,**d**) SEM overview and enlarged view of lattice junctions, respectively.

The ten-layer specimens retained open pores in the top view and a distinct stacked profile in the side view (Figure 7a,b). They were printed through the 150 μm needle with a nominal total height of 1.5 mm. The mean heights of three independently printed specimens were 1.40, 1.42 and 1.375 mm, each measured at five positions using ImageJ. The overall mean height was approximately 1.40 mm (SD = 0.02 mm; n = 3). Retention of both the raised stack and open pores indicates that the setting process supported layer addition in these specimens. SEM images resolved strand connections at a finer scale (Figure 7c,d). The lattice overview showed continuous material across the imaged intersections and distinct spaces between neighboring strands. At higher magnification, the junction surface showed irregular ridges, depressions and smaller pore-like features. The larger lattice openings followed the printed architecture; the finer texture was observed on the surface of the freeze-dried, gold-coated SEM specimen.

### 3.4. Printing Curved Paths and Structures with Variable Height

**Figure 8:**
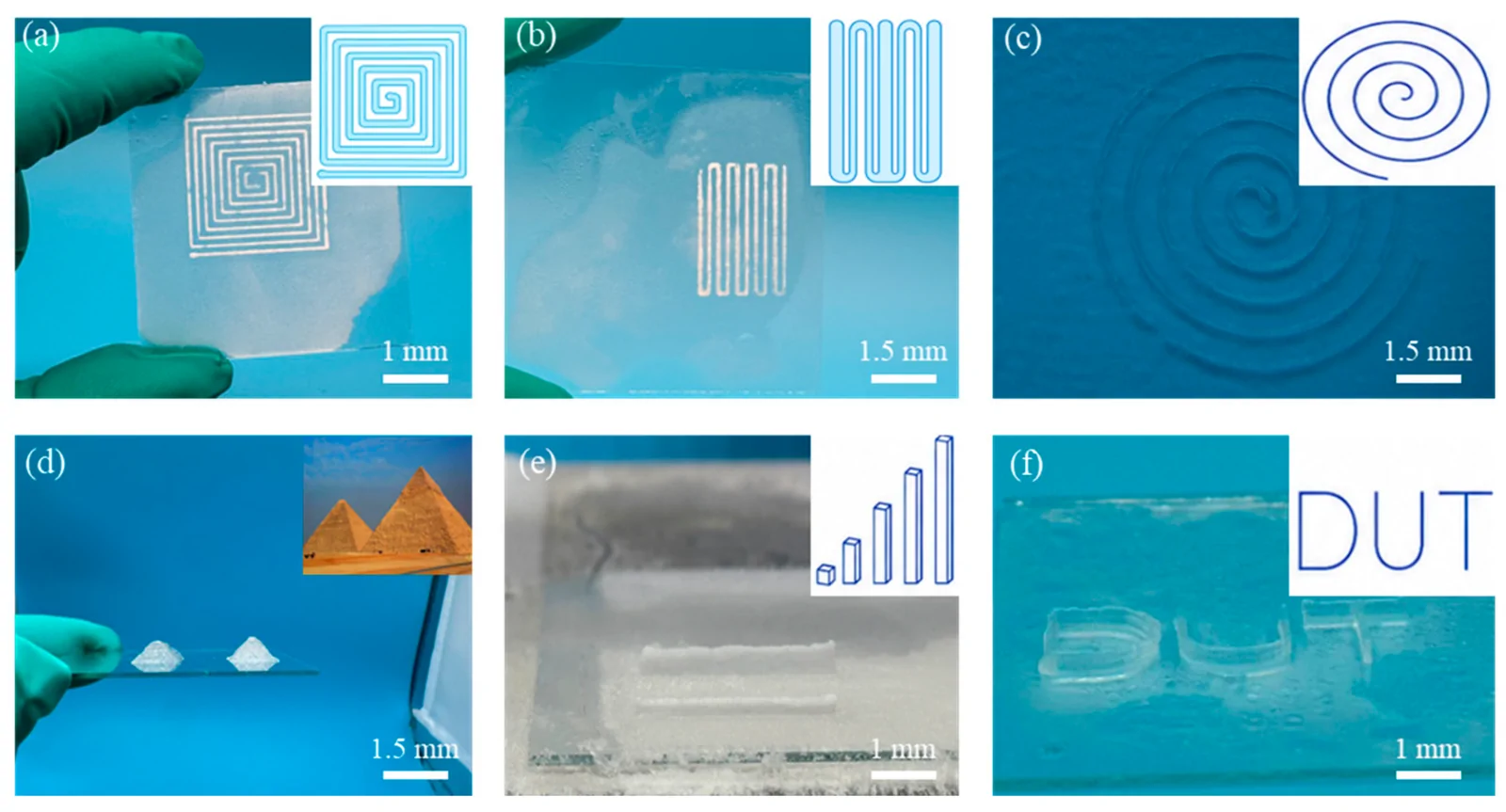
Printed patterns and three-dimensional forms. (**a**) Square spiral. (**b**) Meandering path. (**c**) Circular spiral. (**d**) Pyramidal forms. (**e**) Forms of different heights. (**f**) Lettering.

Beyond regular lattices, the printed structures demonstrated that the process could accommodate abrupt turns, direction reversals, continuous curvature and changes in build height (Figure 8). The square spiral retained gaps between neighboring segments along a recognizable sequence of nested right-angle turns, while the meandering pattern preserved parallel limbs connected by repeated reversals. The circular spiral remained distinct as its path curved around the center. The pyramidal forms narrowed toward their upper regions, illustrating construction with a decreasing cross-section. The specimens of different heights retained distinct upper levels, while raised lettering combined straight and curved strokes with vertical accumulation.

### 3.5. Fabrication of a Meniscus-Shaped Hydrogel Construct

**Figure 9:**
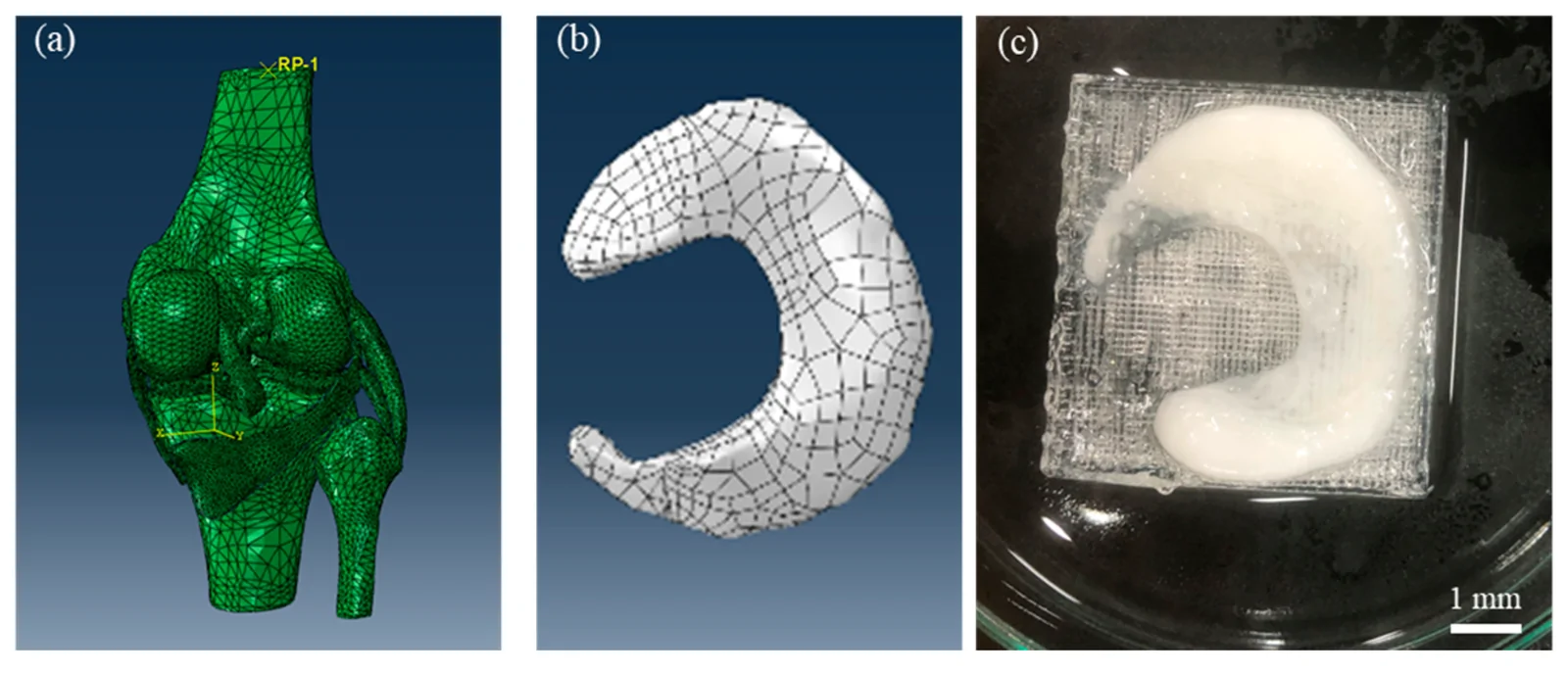
Meniscus-shaped hydrogel fabrication. (**a**) Knee model. (**b**) Meniscus reference geometry. (**c**) Printed hydrogel construct.

The meniscus-shaped construct served as a geometric proof-of-concept, combining a curved external boundary, an internal concavity and separated ends within a single body. Previous studies used protein-releasing polymeric scaffolds for meniscus regeneration in sheep and biomimetic polycaprolactone architectures to examine the relationship between scaffold structure and mechanical response [28,29]. For this fabrication example, the knee model provided anatomical context and the isolated meniscus model supplied the reference crescent outline (Figure 9a,b). The printed hydrogel retained an open C-shaped body, a visible inner concavity and two separated ends (Figure 9c). Its ends were rounded, and the curved body preserved the principal spatial arrangement of the reference shape. Establishing tissue-engineering suitability requires evaluation of the printed architecture’s mechanical response and anisotropy, structural stability after hydration and biological performance.

## 4. Conclusions

This study developed a hydrogel extrusion-printing strategy that combines electrical assistance with substrate cooling to improve the geometric fidelity of PVA/GelMA/alginate scaffolds. Independent control experiments showed that electrical assistance promoted uniform filament formation, whereas substrate cooling restricted lateral spreading and preserved lattice openings. The process produced filaments approximately 152 μm wide and supported open multilayer construction, with ten-layer specimens reaching a mean height of approximately 1.40 mm. Post-print UV crosslinking and freeze–thaw treatment stabilized the fabricated structures, while compression tests on cast hydrogel specimens showed increasing resistance to deformation with additional freeze–thaw cycles. Curved patterns and a meniscus-shaped construct provided a geometric proof of concept for reproducing complex outlines and internal boundaries. Together, the results identify the complementary roles of filament formation, cooling-assisted shape fixation and post-print stabilization in preserving scaffold geometry. The approach supports fabrication of fine, open hydrogel structures with complex geometries under the tested printing conditions.

## Data Availability

The data presented in this study are available on request from the corresponding authors.

## Supplementary Materials

The following supporting information can be downloaded at: https://www.mdpi.com/article/10.3390/polym18202579/s1, Video S1: Filament deposition and shape retention on a cooled substrate..
